# Multisystem embolism in hereditary protein C deficiency with patent foramen ovale: a case report

**DOI:** 10.3389/fcvm.2025.1650286

**Published:** 2025-09-03

**Authors:** Lihua Chen, Jianhua Guan, Yunfeng Ni

**Affiliations:** ^1^ Department of Encephalopathy, The Third Affiliated People’s Hospital of Fujian University of Traditional Chinese Medicine, Fuzhou, China; ^2^ Department of Cerebrovascular Diseases, Fujian Medical University Union Hospital, Fuzhou, China; ^3^ Department of Encephalopathy, The Second Affiliated Hospital of Fujian University of Traditional Chinese Medicine, Fuzhou, China

**Keywords:** hereditary protein C deficiency, patent foramen ovale, right-to-left shunt, multisystem embolism, paradoxical embolism

## Abstract

Hereditary protein C deficiency (HPCD) is a rare autosomal dominant thrombophilia caused by PROC gene mutations, predisposing patients to venous thrombosis. Patent foramen ovale (PFO) with right-to-left shunt (RLS) may facilitate paradoxical embolism, increasing the risk of stroke and pulmonary embolism. However, the coexistence of HPCD and PFO leading to multisystem embolism has not been previously reported. We report a 29-year-old woman presenting with sudden-onset altered consciousness. The patient had no traditional cerebrovascular risk factors other than oral contraceptive use. Imaging revealed basilar artery occlusion, left pulmonary embolism, and bilateral iliac vein thrombosis. Laboratory testing demonstrated reduced protein C activity, and genetic analysis identified a heterozygous pathogenic PROC variant (c.541T>G, p.Phe181Val). Transcranial Doppler bubble study, transesophageal echocardiography (TEE), and agitated saline contrast echocardiography (ASCE) confirmed a PFO with grade 3 RLS. The patient was diagnosed with HPCD combined with PFO. She underwent emergent mechanical thrombectomy, inferior vena cava (IVC) filter placement, subsequent PFO closure, and lifelong rivaroxaban therapy. At 1-year follow-up, she exhibited excellent clinical recovery with complete resolution of symptoms and no evidence of recurrent thromboembolic events. This first-reported case highlights the potential synergistic thrombotic risk of coexisting HPCD and PFO. For patients with unexplained multisystem embolism, thorough evaluation of both conditions is essential, and individualized comprehensive treatment strategies are crucial for a good prognosis.

## 1 Introduction

Hereditary protein C deficiency (HPCD) is a rare autosomal dominant thrombophilic disorder caused by reduced activity of protein C, a critical regulator of coagulation cascade inactivation (1). This defect results in a lifelong hypercoagulable state, predisposing affected individuals to venous thromboembolic events that often manifest with particular severity in younger populations (2). Patent foramen ovale (PFO) is a common congenital cardiac structural abnormality (3). When accompanied by right-to-left shunt (RLS), PFO can serve as a conduit for paradoxical embolism, enabling venous thrombi to bypass the pulmonary circulation and directly enter the arterial circulation, leading to cryptogenic stroke and systemic embolization (4). While HPCD and PFO are both known to increase the risk of thromboembolic events, their synergistic effect in inducing synchronous, life-threatening multi-system thromboembolism involving both arterial and venous systems has not been previously reported.

Here, we present the first documented case of a young female patient developing concurrent arterial and venous thromboembolism mediated by the synergistic pathogenesis of HPCD and PFO. A systematic evaluation was carried out to identify the potential source of the embolus, clarify the pathogenesis, and formulate a targeted treatment strategy.

## 2 Case presentation

A 29-year-old female patient (weight 51 kg, BMI 20.4 kg/m²) presented to the emergency department with acute-onset altered consciousness lasting 7 h. The patient had no traditional cerebrovascular risk factors such as hypertension, diabetes mellitus, or atrial fibrillation but reported one-month use of oral contraceptives prior to symptom onset. Family history was unremarkable. On physical examination, the patient's heart rhythm was regular on auscultation. She was in a shallow coma, with bilateral gaze deviation to the right. She had no voluntary movement in the four limbs, and bilateral Babinski signs were positive. The National Institutes of Health Stroke Scale (NIHSS) score was 24, and the modified Rankin Scale (mRS) score was 5.

**Figure 1:**
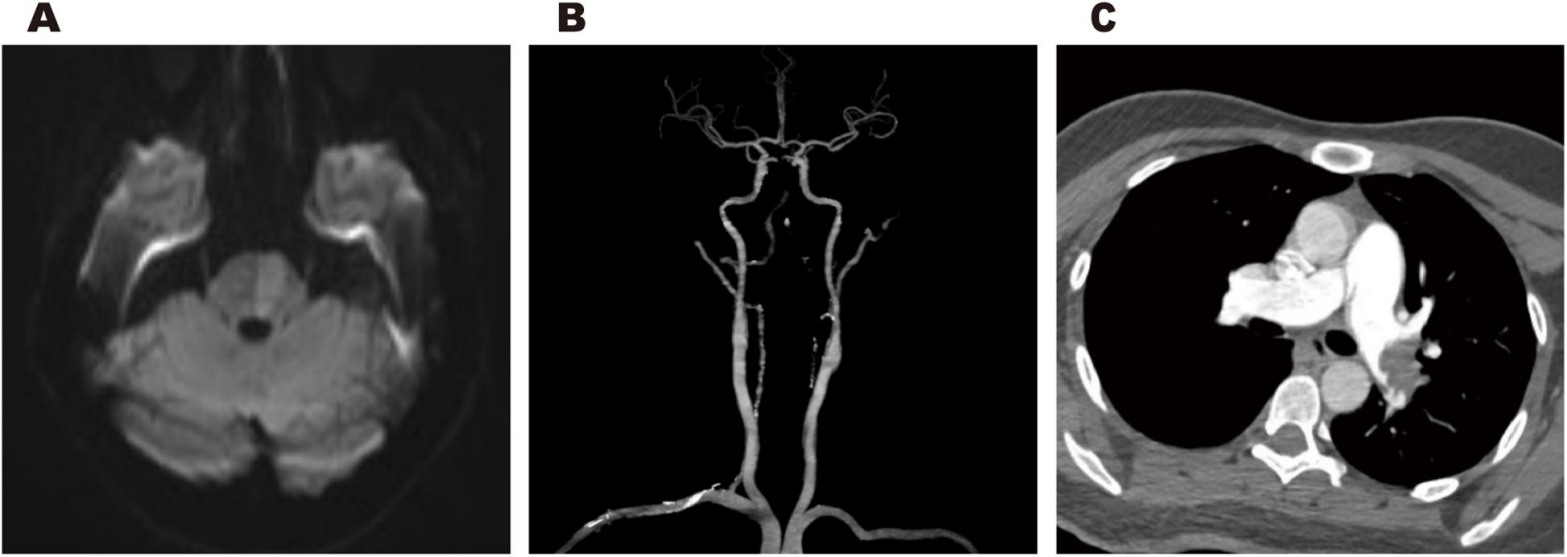
Cranial MRI revealed an acute pontine infarction **(A)**. Head and neck CTA revealed basilar artery occlusion **(B)**, and incidentally showed left pulmonary artery embolism **(C)**.

**Figure 2:**
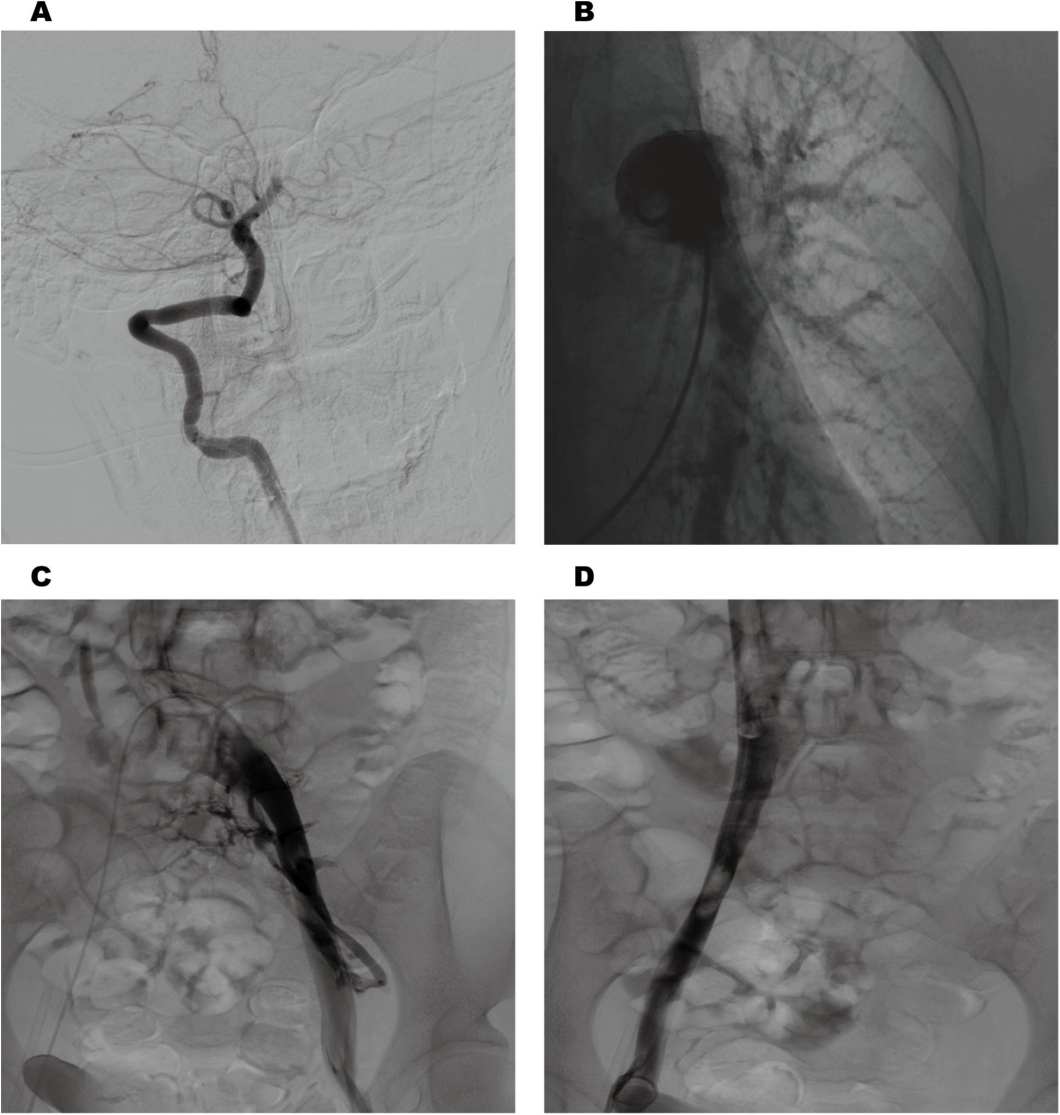
Cerebral angiography revealed interruption of blood flow in the distal basilar artery **(A)** pulmonary angiography revealed left pulmonary artery embolism **(B)**. Antegrade venography of the lower extremity deep veins revealed bilateral iliac vein thrombosis **(C,D)**.

**Figure 3:**
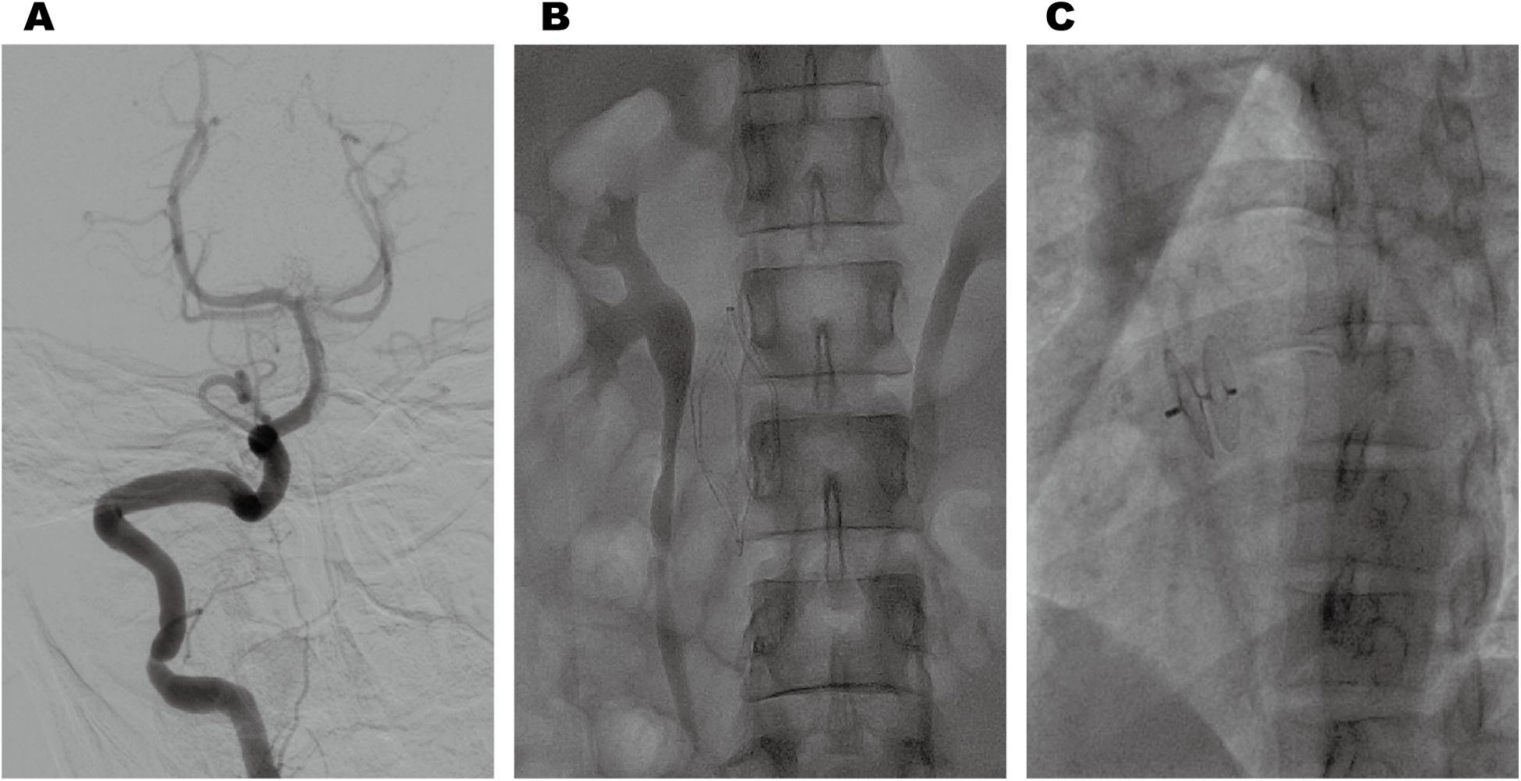
Post-thrombectomy imaging revealed normal anterograde blood flow in the basilar artery **(A)**. Postoperative changes following inferior vena cava filter implantation **(B)**. Post-procedural imaging revealed appropriate positioning of the PFO closure device **(C)**.

Cranial magnetic resonance imaging (MRI) revealed an acute pontine infarction (Figure 1A). Head and neck computed tomography angiography (CTA) showed basilar artery occlusion (Figure 1B) and incidentally detected left pulmonary artery embolism (Figure 1C), suggesting the possibility of multisystem embolism. Further digital subtraction angiography (DSA) of the whole brain confirmed interruption of blood flow in the distal basilar artery (Figure 2A). Pulmonary angiography revealed left pulmonary artery embolism (Figure 2B). Antegrade venography of the lower extremity deep veins showed bilateral iliac vein thrombosis (Figures 2C,D). The patient underwent emergency mechanical thrombectomy of the basilar artery and placement of an inferior vena cava filter (Figure 3B). Post-operatively, imaging evaluation showed the basilar artery was patent (Figure 3A), and the patient was transferred to the neurology department for further treatment.

After excluding hemorrhagic complications via 24-h post-operative CT scan, therapeutic enoxaparin (4,000 IU every 12 h) was initiated for multisystem thromboembolism. We performed comprehensive testing including homocysteine, thyroid function tests, hemoglobin A1c, antinuclear antibody (ANA), anti-double stranded DNA (dsDNA), anticardiolipin antibodies (IgG/IgM), anti-β2-glycoprotein 1 antibodies (IgG/IgM), anti-cyclic citrullinated peptide (CCP) antibody, anti-keratin antibody (AKA), lupus anticoagulant (LAC) testing, hepatitis B serology, hepatitis C antibody, syphilis antibody, HIV antibody, CEA, AFP, CA125, CA153, CA199, computed tomography scan of the chest and complete abdominal ultrasound to exclude metabolic disorders, autoimmune diseases, malignancies, and infectious diseases as potential causes of thrombosis. Coagulation function tests showed markedly elevated D-dimer (>35.2 mg/L) with reduced protein C activity (59%; reference range 65%–135%). Protein S and thrombin levels were within normal limits. Thrombophilia genetic panel identified a heterozygous pathogenic variant in the PROC gene (c.541T>G, p.Phe181Val), which was also confirmed in her asymptomatic father, confirming hereditary protein C deficiency (HPCD). After one week of anticoagulation therapy, follow-up color Doppler ultrasound of the bilateral lower extremity veins showed recanalization changes in the bilateral common and external iliac veins, with no obvious abnormalities in the bilateral deep veins of the lower extremities.

**Figure 4:**
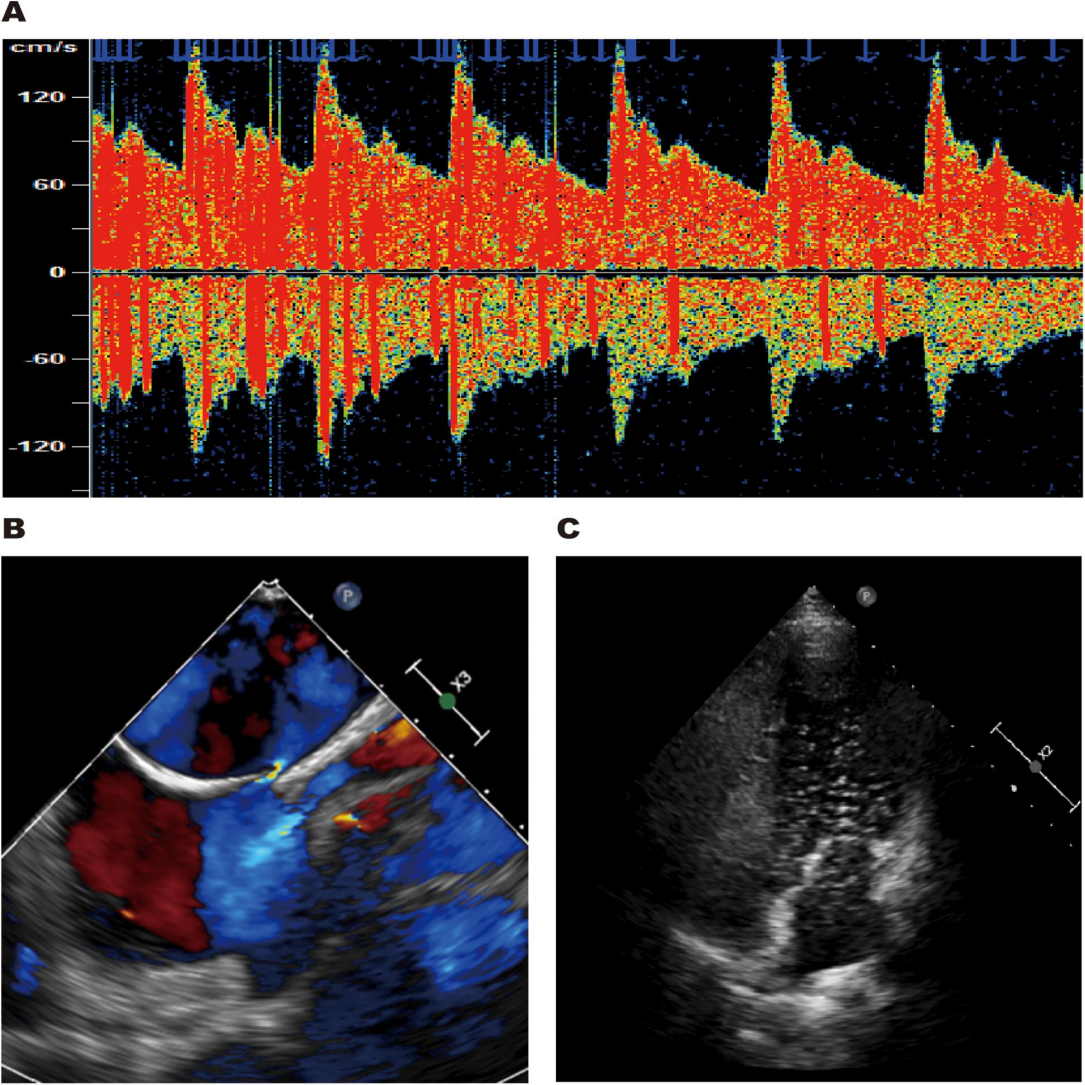
TCD bubble study revealed a grade 3 right-to-left shunt during the Valsalva maneuver **(A)**. TEE showed the primary and secondary septa of the atrial septum were separated by approximately 2.7 mm **(B)**. ASCE revealed large microbubble passage into the left atrium during the Valsalva maneuver **(C)**.

The patient had no traditional cerebrovascular risk factors, and her RoPE score was 9. Therefore, we conducted a comprehensive cardiac evaluation to rule out the possibility of paradoxical embolism. Doppler bubble study demonstrated >25 microbubbles (Grade 3 right-to-left shunt) during the Valsalva maneuver, displaying the characteristic “shower-curtain” pattern (Figure 4A). Further transesophageal echocardiography (TEE) showed the primary and secondary septa of the atrial septum were separated by approximately 2.7 mm, indicating a patent foramen ovale (Figure 4B). Agitated saline contrast echocardiography (ASCE) showed moderate microbubble passage into the left atrium at rest, markedly increasing during the Valsalva maneuver (Figure 4C), confirming significant intracardiac right-to-left shunting.

Based on these examinations, we confirmed that the patient with HPCD had paradoxical embolism related to PFO. After clinical stabilization, the patient underwent successful percutaneous closure of the PFO using a specific closure device (PF2525) (Figure 3C). Anticoagulation was transitioned from heparin to long-term rivaroxaban with a recommendation for lifelong therapy. At 1-year follow-up, the patient achieved complete neurological recovery (NIHSS 0, mRS 0) without recurrent thromboembolic events.

## 3 Discussion

No prior studies have documented multisystem embolism arising from the concurrent presence of HPCD and PFO. HPCD is caused by mutations in the PROC gene encoding protein C (5). Protein C is a vitamin K-dependent plasma glycoprotein that functions as a critical physiological anticoagulant by regulating thrombin generation through the proteolytic inactivation of coagulation factors Va and VIIIa. PROC gene mutations lead to abnormalities in the synthesis, function, or stability of protein C, resulting in reduced anticoagulant activity of the blood and an increased risk of thrombosis (6). While HPCD typically manifests with venous thromboembolism, this case presented an unusual arterial-dominant phenotype, with initial manifestation as acute cerebral arterial occlusion. This exceptionally rare presentation in isolated HPCD strongly suggests paradoxical embolism.

The patient was found to have a coexisting PFO through TCD bubble study and transesophageal echocardiography (TEE). PFO is present in about 25% of the general population, and most patients are asymptomatic due to small shunt volumes. However, when there is a source of venous thrombi, the risk of paradoxical embolism through a PFO increases significantly. Under special inducements such as severe coughing, the Valsalva maneuver, or straining during defecation, right atrial pressure rises instantaneously, triggering right-to-left shunt, which allows venous thrombi to bypass the pulmonary circulation and enter the arterial circulation directly, ultimately leading to paradoxical embolism (7). This mechanism is widely considered an important cause of paradoxical embolism, especially in young people (8).

This case highlights the synergistic pathogenic effects of three concomitant risk factors: hereditary thrombophilia, acquired prothrombotic elements, and cardiac structural abnormalities. The thrombotic cascade in this case originates from the profound anticoagulation impairment caused by HPCD. Genetically deficient protein C activity fails to adequately regulate thrombin generation, resulting in uncontrolled propagation of clot formation that manifested clinically as extensive iliac vein thrombosis. The hypercoagulable state was further exacerbated by oral contraceptive use through multiple synergistic mechanisms. It upregulated the levels of procoagulant factors while simultaneously downregulating protein C activity. This pharmacological impact worked in tandem with her underlying genetic defect of protein C deficiency, further intensifying the hypercoagulable state (9). This pharmacological potentiation of coagulation worked in concert with the patient's underlying PROC gene mutation, creating a particularly severe thrombogenic milieu that predisposed to the development of multi-system thromboembolism. The *in situ* thrombi formed within the iliac veins then spread through two distinct pathways: some emboli traveled via the right heart circulation into the pulmonary arterial system, while others passed through the PFO into the systemic circulation. Ultimately, this led to the simultaneous occurrence of cerebral arterial embolism and pulmonary embolism. HPCD provides the thrombogenic substrate, while the PFO acts as the anatomical enabler for multisystem embolism. This pattern of multisystem embolism is relatively rare in clinical practice and has characteristic pathophysiological mechanisms.

The patient received a staged comprehensive treatment plan. Given the >80% mortality in untreated basilar occlusions, mechanical thrombectomy remains the cornerstone of acute management. In the acute phase, mechanical thrombectomy of the basilar artery was performed for the life-threatening basilar artery occlusion, which aligned with current stroke guidelines for posterior circulation large-vessel occlusion (10). At the same time, an inferior vena cava filter was placed in the inferior vena cava. This intervention bridged the patient to definitive therapy while mitigating fatal embolism risk. Recent randomized controlled trials have shown that PFO closure in such high-risk patients reduces stroke recurrence compared with drug therapy alone (11, 12). Based on the RoPE score assessment, the patient was stratified as high risk for PFO-related paradoxical embolism (13). Comprehensive workup excluded other potential embolic sources, confirming the PFO as the anatomical substrate facilitating paradoxical embolization. The patient underwent PFO closure after neurological stabilization. Despite successful PFO closure, lifelong anticoagulation remained imperative due to the persistent prothrombotic state inherent to HPCD. This dual therapeutic approach—combining anatomical shunt correction with sustained pharmacological anticoagulation—offers optimal protection against both paradoxical embolism and recurrent venous thromboembolism. Previous studies have demonstrated that traditional vitamin K antagonists (VKAs) may exacerbate the depletion of vitamin K-dependent proteins in patients with HPCD, potentially triggering complications such as skin necrosis (14). In contrast, direct oral anticoagulants (DOACs) such as rivaroxaban specifically inhibit factor Xa without interfering with the protein C system, potentially making them a more suitable option for HPCD patients (15, 16). The patient chose rivaroxaban for long-term anticoagulation, and no thrombotic recurrence occurred during the follow-up period, supporting the effectiveness of this strategy. However, current evidence on DOACs for HPCD primarily derives from subgroup analyses within larger thrombophilia studies. A prospective cohort study involving 255 patients with inherited thrombophilia—including 21 cases of HPCD—demonstrated that DOACs exhibit efficacy comparable to vitamin K antagonists in preventing venous thromboembolism (VTE) recurrence. However, the DOACs group showed a higher overall bleeding risk, with a significant increase in clinically relevant non-major bleeding events. Notably, the DOACs group had a significantly lower risk of VTE recurrence within 2 years after discontinuation (17). These findings suggest that DOACs may represent a reasonable option for selected HPCD patients, particularly those concerned about the risk of recurrence after cessation. Nevertheless, the associated bleeding risks must be carefully weighed. It is important to emphasize that these conclusions are based on subgroup analyses with small sample sizes and thus require validation through large-scale studies specifically designed for the HPCD population.

This case indicates that in the presence of concurrent HPCD and PFO, the hypercoagulable state and the paradoxical embolism mechanism jointly contribute to an augmented risk of multi-system embolism. Clinicians must remain highly vigilant in cases where hereditary anticoagulant deficiency coexists with PFO. Comprehensive laboratory examinations, including thrombophilia screening and genetic testing, as well as a comprehensive cardiac evaluation, especially the detection of right-to-left shunt, are crucial for clarifying the etiology. Individualized comprehensive treatment strategies, including acute-phase vascular recanalization, PFO closure, and long-term anticoagulation therapy, may be the key to improving the prognosis of such patients.

## Data Availability

The original contributions presented in the study are included in the article/Supplementary Material, further inquiries can be directed to the corresponding author.
